# Exploring the causal relationship between immune cell and all-cause heart failure: a Mendelian randomization study

**DOI:** 10.3389/fcvm.2024.1363200

**Published:** 2024-06-13

**Authors:** Jixu Li, Liangliang Liu, Qiuyan Luo, Weiyue Zhou, Yao Zhu, Weimin Jiang

**Affiliations:** Department of Cardiology, Affiliated Hospital of Nanjing University of Chinese Medicine, Nanjing, Jiangsu, China

**Keywords:** immune cell, all-cause heart failure, Mendelian randomization, single nucleotide polymorphism, causal effect

## Abstract

**Background and objectives:**

Heart failure (HF) is a disease with numerous genetic and environmental factors that affect it. The results of previous studies indicated that immune phenotypes are associated with HF, but there have been inconclusive studies regarding a causal relationship. Therefore, Mendelian randomization (MR) analyses were undertaken to confirm the causal connections between immune phenotypes and HF, providing genetic evidence supporting the association of immune cell factors with HF risk.

**Methods:**

We selected instrumental variables that met the criteria based on data from the results of genome-wide association studies (GWAS) of immune phenotype and all-cause HF. An evaluation of the causal association between 731 immune cell factors and HF risk was carried out using the inverse variance weighted (IVW), MR-Egger regression (MR-Egger), and weighted median (WM) analysis methods. To determine the horizontal pleiotropy, heterogeneity, and stability of the genetic variants, the MR-Egger intercept test, Cochran's *Q* test, MR-PRESSO, and leave-one-out sensitivity analysis were performed.

**Results:**

MR principal method (IVW) analysis showed that a total of 38 immune cell-related factors were significantly causally associated with HF. Further analyses combining three methods (IVW, MR-Egger and WME) showed that six exposure factors significantly associated with heart failure, as shown below. The effect of Dendritic cell Absolute Count, CD62l- CD86+ myeloid Dendritic cell Absolute Count, CD62l- CD86+ myeloid Dendritic cell% Dendritic cell, CD39+ CD8+ T cell% CD8+ T cell, CD3 on Central Memory CD4+ T cell on heart failure was positive. Whereas, a reverse effect was observed for CD14+ CD16+ monocyte% monocyte.

**Conclusion:**

We investigated the causal relationship between immune phenotypes and all-cause HF. According to the results, Dendritic cell Absolute Count, CD62l- CD86+ myeloid Dendritic cell Absolute Count, CD62l- CD86+ myeloid Dendritic cell% Dendritic cell, CD39+ CD8+ T cell% CD8+ T cell, CD3 on Central Memory CD4+ T cell aggravate HF, and the risk of HF is decreased by CD14+ CD16+ monocyte% monocyte. These phenotypes may serve as new biomarkers, providing new therapeutic insights for the prevention and treatment of all-cause HF.

## 1 Introduction

In general, heart failure (HF) is caused by various structural or functional disorders of the heart that result in impaired ventricular filling and (or) ejection capacity, represented as dyspnea, limitation of physical activity and fluid retention (1). At present, approximately 64 million people are thought to suffer from HF worldwide, 20% of total cardiovascular cases, and have a prevalence of around 1%–12% (2–4). In 2019, a meta-analysis involving 1.5 million chronic heart failure patients revealed that the comprehensive survival rates for these patients at one year, two years, five years, and ten years were 86.5%, 72.6%, 56.7%, and 34.9%, respectively (5). HF has the characteristics of high morbidity, high hospitalization rate, and high mortality, and it accounts for a large economic and societal burden. Therefore, early diagnosis and treatment of HF to improve the condition of patients with HF and to reduce morbidity and mortality has become a significant public health concern around the world (6).

With increasing research into the pathophysiological mechanisms of HF, evidence is mounting that immune activation is involved in the process of HF. When the heart is damaged, various stimuli lead to the activation of effector immune cells, penetrating the vascular wall. Different subgroups of immune cells release various cytokines, including interleukin-6, interleukin-17, interleukin-10, tumor necrosis factor-alpha, and interferon-gamma. These cytokines target the regulation of vascular aging, degradation of elastic lamina, and the process of myocardial fibrosis. While HF generates cellular damage and structural remodeling, it can secrete related cytokines and substances, further intensifying this immune-inflammatory response. This, in turn, leads to additional damage to the body's heart and other organ tissues, creating a chronic malignant cycle. Consequently, this accelerates the progression of HF and leads to an adverse prognosis (7). In relation to HF, immune phenotypes have been shown to influence host immunity (8–10). Some studies suggest in the onset of HF, activation of NFKb and NLRP3 inflammasome triggers downstream production of IL-1b and IL-6, while clonal hematopoiesis mediated by TET2 is recognized as an accelerating factor in HF deterioration (11, 12). In Houssari M's investigation into the influence of infiltrating T cells on post-infarction cardiac lymphatic remodeling, the findings indicate that CD4 and CD8T cells, to a certain extent, effectively suppress the generation of cardiac lymphatic vessels through interferon-gamma, consequently slowing down cardiac remodeling and myocardial fibrosis following myocardial infarction (13). However, there are many factors that may affect the results of current research, including sample size, limitations in study design, and confounding variables. To date, there is still no consensus on the correlation between immune phenotypes and overall HF.

In recent years, genomics-wide association studies (GWAS) have provided the opportunity to uncover HF's genetic background, the linkage disequilibrium score regression (LDSC) and genomic structural equation modeling (Genomic SEM) can evaluate genetic correlations and partial correlations based on aggregated-level data (14, 15). HF is a complex disorder with an estimated heritability of around 26%. The Mendelian randomization (MR) method uses measured genetic variants as instrumental variables for exposures to infer the causation of the effects of exposures (16). Large-scale GWAS have identified numerous single nucleotide polymorphism (SNP) loci, providing a wealth of genetic variation instrumental variables for MR analyses, effectively reducing bias in causal relationship estimates (17). At the same time, because genotype precedes disease and is primarily independent of postnatal habits and environment, the theory ensures that confounding factors are balanced between different genetic variants. Thus, an analysis of MR has been used to determine potentially causal relationships between exposures and diseases (18–20).

In this study, all the current GWAS data related to 731 immune cells were collected and the causal effects of all immune cells and related factors on all-cause HF were assessed using a two-sample MR approach, which systematically screened out immune cells that may contribute to all-cause HF and provided new ideas for preventing and treating HF from an immune perspective.

## 2 Materials and methods

### 2.1 Study design

In this study, we systematically analyzed the potential causal effects of 731 immune cell phenotypes associated with all-cause HF using a two-sample MR analysis and evaluated the reliability of the results.

### 2.2 Data sources for exposure and outcomes

The summary data of the GWAS for immune cells is sourced from IEU Open GWAS (https://gwas.mrcieu.ac.uk/). The total sample size is 2,919 cases, with a total of 14,849,624 SNPs (21). The summary data regarding the GWAS for overall HF originates from the 9th edition of research data conducted by the FinnGen Consortium. This comprehensive study involves 376,233 Finnish adult participants, comprising 26,872 cases and 349,361 controls, encompassing a total of 5,747,754 SNPs (https://r9.finngen.fi/pheno/I9_HEARTFAIL_ALLCAUSE) (22). Data aggregated from publicly available sources was used in the study and, therefore, did not require ethical approval and consent for participation.

### 2.3 Instrument selection

In MR, genetic variants must meet three core assumptions of an instrumental variable (23). (1) The relevance assumption: genetic variants used as instrumental variables should be associated with the risk factor. (2) Independence assumption: the selected SNPs do not interact with other variables. (3) Exclusionary restriction assumption: instrumental variables affect all factors—the only factors contributing to HF are immune cell exposure factors and not alternative pathways.

**Figure 1:**
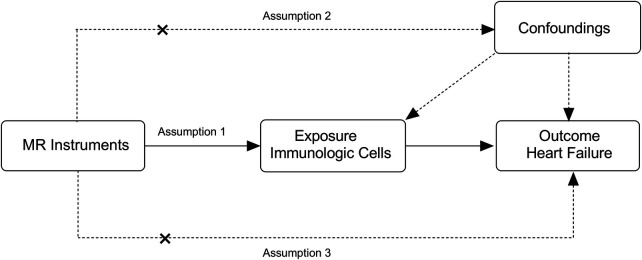
Overview of the overall MR design. (1) Assumption 1: the relevance assumption, genetic variants used as instrumental variables should be associated with the risk factor. (2) Assumption 2: the independence assumption, the selected genetic variants do not interact with other variables. (3) Assumption 3: the exclusion restriction assumption, instrumental variables affect all—cause HF only through immune cell exposure factors and not through additional pathways.

To meet the above assumptions, we developed specific screening criteria for the instrumental variables. We analyzed the linkage disequilibrium of each risk factor based on the 1,000 genomes reference panel (European population). In the next step, a set of parameters was used to LD-clump the processed summary files (PLINK software, *p*1 = 1 × 10-5, *r*^2^ = 0.1, and distance 500 kb). Finally, we removed any outliers that may have been affected by pleiotropic bias using MR—PRESSO and Radial MR (24). The *F* statistic was approximated to determine whether the instrumental variable had a weak bias. Weak instrumental variables were defined as those with an *F* statistic less than 10 (25) (Figure 1).

In order to remove SNPs associated with confounding factors and outcomes of HF, we manually screened and deleted them using PhenoScanner (http://www.phenoscanner.medschl.cam.ac.uk/) (26).

### 2.4 MR analysis

As a primary analysis, we used IVW for its efficiency in estimating the causal effect of immune phenotypes on the HF (27), combining the WM approach and MR-Eggerwas used as complementary analyses (28, 29). The joint analysis of the above multiple MR methods can test the stability and reliability of the association under different assumptions.

### 2.5 Sensitivity analysis

We performed heterogeneity tests, pleiotropy tests, and leave-one-out tests during the sensitivity analysis. The transverse pleiotropy was tested using the MR-Egger intercept. In the MR-Egger analysis with an intercept of 0.05, there probably isn't a horizontal pleiotropic pathway (30). We evaluated SNP heterogeneity using Cochran' s *Q* test. The fixed-effects model was applied for SNPs with no evidence of heterogeneity between studies (Cochran's *Q* test *P* < 0.05), and the random effects model was applied for SNPs exhibiting heterogeneity between studies (Cochran's *Q* test *P* > 0.05) (31). For the detection of outliers, we used the MR-pleiotropy residual sum and outlier (MR—PRESSO) test. If outliers were present, they were removed, and we re-evaluated the MR causal estimates. Additionally, we conducted a leave-one-out analysis to test whether one SNP had a significant effect (32). Odds ratios (ORs) and 95% confidence intervals (CIs) are presented to show the association between the potential associated factors and HF. A significant difference was considered when *P* was <0.05.

### 2.6 Reverse MR analysis

To assess whether HF has a causal effect on the levels of immune cells in the body, the MR mentioned above analysis method was employed. In this approach, systemic HF was considered as the exposure, and individual immune cell phenotypes were separately examined as outcomes in a reverse MR analysis. Two-sample MR and MR-PRESSO packages (version 1.0 and 0.5.4) of the R program (version 4.3.1) were used for statistical analysis (33).

## 3 Results

### 3.1 Instrumental variable selection results

This study utilized GWAS data for 731 immune cell phenotypes and associated factors as the focus of investigation. Employing criteria including a significance threshold of *P* < 1 × 10-05 and linkage disequilibrium analysis, a total of 38 immune cell phenotypes were identified through MR analysis, with an average *F*-statistic exceeding 10 (Table 1).

**Table 1:** Compilation of 38 immune cell phenotypes.

Category	Phenotypes	Abbreviation	SNPs	GWAS ID
B cell	IgD+ B cell Absolute Count	IgD+ B cell Absolute Count	15	ebi-a-GCST90001400
	IgD- CD27- B cell %lymphocyte	IgD- CD27- B cell %lymphocyte	22	ebi-a-GCST90001433
	CD19 on IgD- CD24- B cell	CD19 on IgD- CD24- B cell	24	ebi-a-GCST90001731
Dendritic cell	Myeloid Dendritic Cell Absolute Count	MDC Absolute Count	53	ebi-a-GCST90001458
	Dendritic Cell Absolute Count	DC Absolute Count	44	ebi-a-GCST90001461
	CD62l- plasmacytoid Dendritic Cell Absolute Count	CD62l- pDC Absolute Count	32	ebi-a-GCST90001470
	CD62l- CD86+ myeloid Dendritic Cell Absolute Count	CD62l- CD86+ MDC Absolute Count	83	ebi-a-GCST90001472
	CD62l- CD86+ myeloid Dendritic Cell %Dendritic Cell	CD62l- CD86+ MDC %DC	86	ebi-a-GCST90001473
	CD62l on CD62l+ myeloid Dendritic Cell	CD62l on CD62l+ MD Cell	23	ebi-a-GCST90001831
T cell	CD25++ CD4+ T cell %T cell	CD25++ CD4+ T cell %T cell	24	ebi-a-GCST90001506
	CD25++ CD45RA- CD4 not regulatory T cell Absolute Count	CD25++ CD45RA- CD4 not Treg Absolute Count	25	ebi-a-GCST90001510
	Terminally Differentiated CD4+ T cell Absolute Count	TD CD4+ T cell	18	ebi-a-GCST90001545
	T cell Absolute Count	T cell Absolute Count	21	ebi-a-GCST90001603
	CD8+ Natural Killer T Absolute Count	CD8+ NKT cell Absolute Count	29	ebi-a-GCST90001630
	CD39+ CD8+ T cell %CD8+ T cell	CD39+ CD8+ T cell %CD8+ T cell	196	ebi-a-GCST90001671
	CD3 on Central Memory CD4+ T cell	CD3 on CM CD4+ T cell	60	ebi-a-GCST90001841
	CD3 on CD45RA- CD4+ T cell	CD3 on CD45RA- CD4+ T cell	64	ebi-a-GCST90001845
	CD3 on Central Memory CD8+ T cell	CD3 on CM CD8+ T cell	48	ebi-a-GCST90001846
	CD3 on Natural Killer T	CD3 on NKT	21	ebi-a-GCST90001848
	CD3 on T cell	CD3 on T cell	56	ebi-a-GCST90001851
	CD3 on CD39+ activated CD4 regulatory T cell	CD3 on CD39+ activated CD4 Treg	72	ebi-a-GCST90001854
	CD3 on secreting CD4 regulatory T cell	CD3 on secreting CD4 Treg	64	ebi-a-GCST90001855
	CD3 on activated & secreting CD4 regulatory T cell	CD3 on activated & secreting CD4 Treg	77	ebi-a-GCST90001857
	CD3 on CD45RA+ CD4+ T cell	CD3 on CD45RA+ CD4+ T cell	88	ebi-a-GCST90001858
	CD3 on CD39+ CD4+ T cell	CD3 on CD39+ CD4+ T cell	76	ebi-a-GCST90001860
	CD3 on CD28+ CD4-CD8- T cell	CD3 on CD28+ CD4- CD8- T cell	14	ebi-a-GCST90001862
	CD3 on CD4 regulatory T cell	CD3 on CD4 Treg	67	ebi-a-GCST90001868
	HVEM on Effector Memory CD4+ T cell	HVEM on CD4+ TEM	22	ebi-a-GCST90001878
	CD28 on CD39+ activated CD4 regulatory T cell	CD28 on CD39+ activated CD4 Treg	36	ebi-a-GCST90001886
Monocyte	CD14+ CD16+ monocyte %monocyte	CD14+ CD16+ monocyte %monocyte	66	ebi-a-GCST90001585
	CD45 on CD14+ monocyte	CD45 on CD14+ monocyte	18	ebi-a-GCST90001909
	FSC-A on HLA DR+ Natural Killer	FSC-A on HLA DR+ NK	46	ebi-a-GCST90001970
	CCR2 on monocyte	CCR2 on monocyte	43	ebi-a-GCST90002017
	CD45 on CD33+ HLA DR+ CD14-	CD45 on CD33+ HLA DR+ CD14-	19	ebi-a-GCST90002042
	CD33dim HLA DR+ CD11b- %CD33dim HLA DR+	CD33dim HLA DR+ CD11b- %CD33dim HLA DR+	30	ebi-a-GCST90001528
MDSCs	CD11b on Granulocytic Myeloid-Derived Suppressor Cells	CD11b on G-MDSC	3	ebi-a-GCST90002092
	Granulocytic Myeloid-Derived Suppressor Cells Absolute Count	MDSCs	27	ebi-a-GCST90001524
	CD34 on Hematopoietic Stem Cell	CD34 on HSC	19	ebi-a-GCST90001870

### 3.2 Main results of the MR analysis

**Figure 2:**
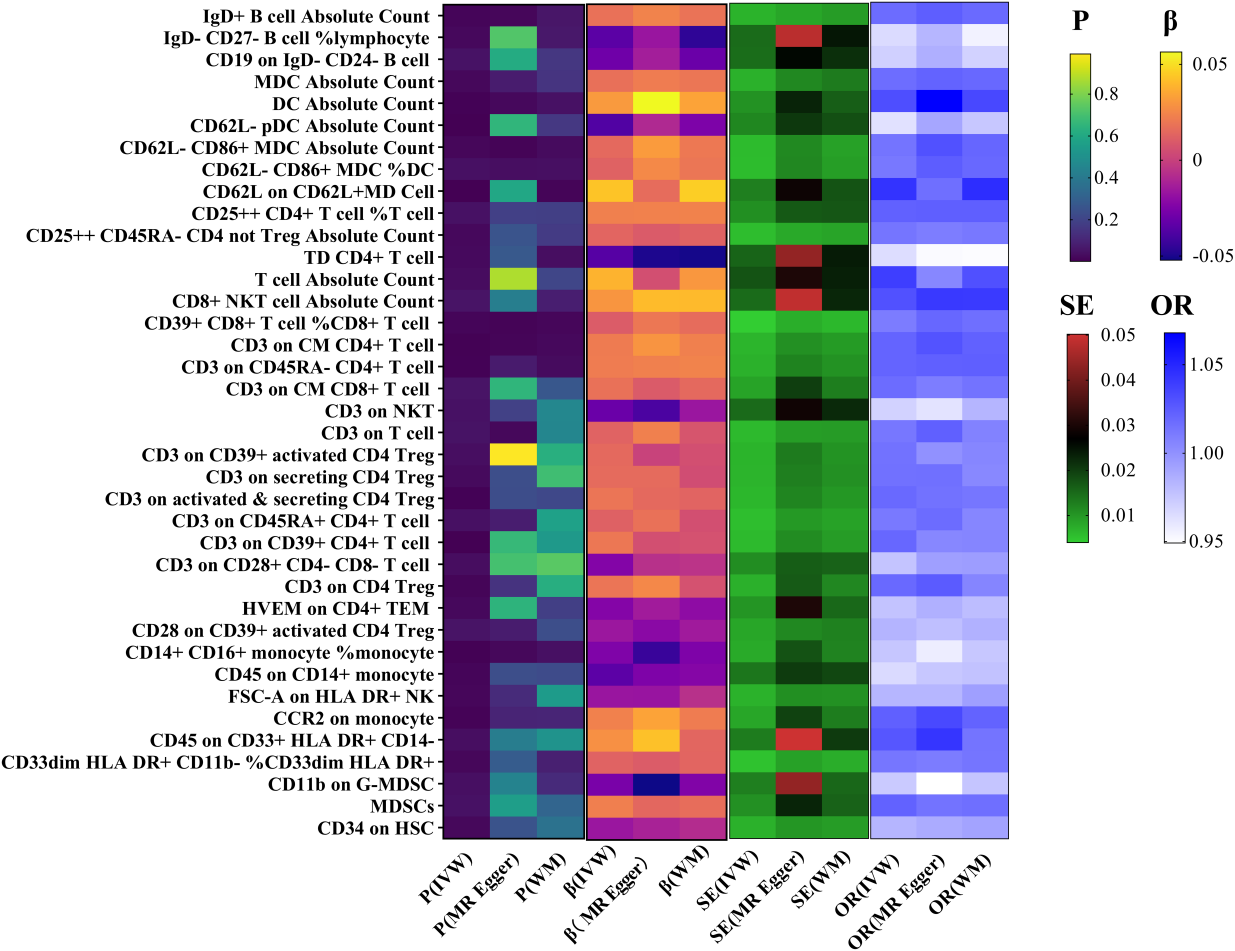
Association between immune cells and HF.

In the primary analysis using the IVW method, a positive correlation was observed between 25 immune cell-related factors and the risk of HF (OR > 1). Conversely, 13 immune cell-related factors showed a negative correlation (OR < 1) with the incidence of HF (Figure 2).

### 3.3 Combined results of the three methods in MR analysis

In the combined analysis using IVW, MR-Egger regression, and WM, Dendritic cell Absolute Count, CD62l- CD86+ myeloid Dendritic cell Absolute Count, and CD62l- CD86+ myeloid Dendritic Cell% Dendritic cell exhibited potential positive causal effects on HF (0.001 < *P* < 0.05). Specifically, an increase in Dendritic cell Absolute Count levels is associated with an approximately 3% higher risk of HF incidence (IVW: OR = 1.03, 95% CI: 1.01–1.06). Similarly, CD62l- CD86+ myeloid Dendritic cell Absolute Count (IVW: OR = 1.013, 95% CI: 1.00–1.03) and CD62l- CD86+ myeloid Dendritic cell% Dendritic cell (IVW: OR = 1.01, 95% CI: 1.00–1.02) are also positively correlated with the onset of HF.

**Figure 3:**
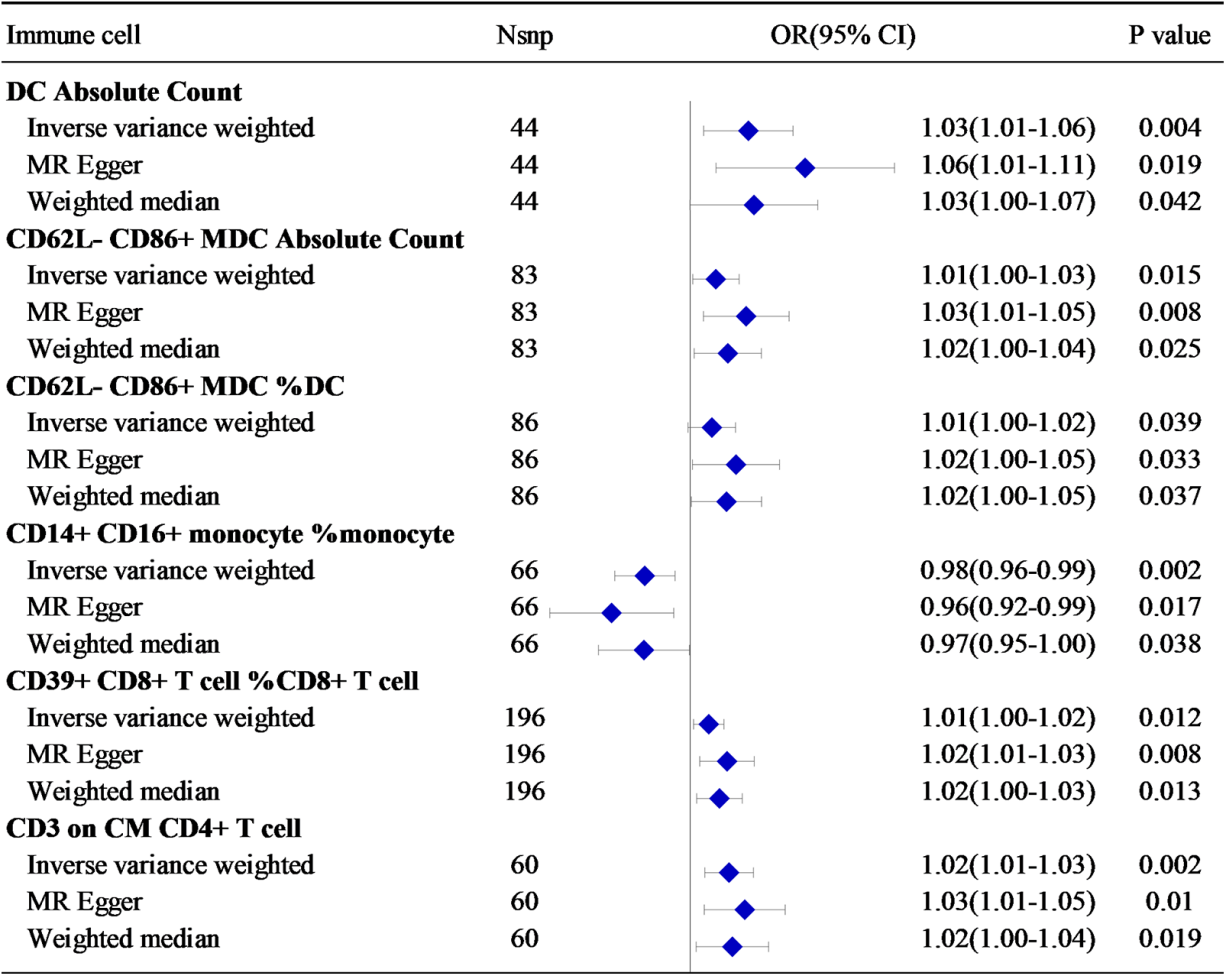
The relationship between six immune cell-related factors and HF.

For HF, there is a significant positive causal effect for CD39+ CD8+ T cell% CD8+ T cell [OR = 1.01, 95% CI (1.00–1.02), *P* = 0.039], as well as CD3 on Central Memory CD4+ T cell [OR = 1.02, 95% CI (1.01–1.03), *P* = 0.002] (0.001 < *P* < 0.05). On the contrary, there is a negative correlation between CD14+ CD16+ monocyte% monocyte [OR = 0.98, 95% CI (0.96–0.99), *P* = 0.002] and the incidence risk of HF. In other words, for each increase of one standard deviation in CD14+ CD16+ monocyte% monocyte, the average risk of developing systemic HF may decrease by 2% (Figure 3).

### 3.4 Reverse MR analysis

The findings from the reverse MR study suggest that systemic HF does not appear to be a significant factor causing alterations in immune cells. Additionally, the likelihood ratio, MR-Egger intercept, and WM did not reveal a causal link between these variables.

### 3.5 Sensitivity analysis

Using MR-Eggerto assess horizontal pleiotropy between SNPs and outcomes, the results indicate that Dendritic cell Absolute Count (intercept *P* = 0.136), CD62l- CD86+ myeloid Dendritic cell Absolute Count (intercept *P* = 0.159), CD62l- CD86+ myeloid Dendritic cell% Dendritic cell (intercept *P* = 0.075), CD14+ CD16 + monocyte% monocyte (intercept *P* = 0.176), CD39+ CD8+ T cell% CD8+ T cell (intercept *P* = 0.156), and CD3 on Central Memory CD4+ T cell (intercept *P* = 0.947) show no evidence of horizontal pleiotropy with respect to systemic HF (*P* > 0.05) (Table 2).

**Table 2:** MR sensitivity analyses.

Immune phenotypes	Cochran's Q statistic			MR-Egger regression			MR-PRESSO	
	Method	Cochran's Q	*P* value	Egger-intercept	SE	*P* value	Test	*P* value
DC Absolute Count	MR-Egger	52.364	0.131	−0.006	0.005	0.202	57.119	0.136
	IVW	54.457	0.113					
CD62l- CD86+ MDC Absolute Count	MR-Egger	92.177	0.186	−0.006	0.003	0.101	97.168	0.159
	IVW	95.316	0.149					
CD62l- CD86+ MDC Absolute Count	MR-Egger	103.132	0.077	−0.004	0.003	0.196	107.231	0.075
	IVW	105.216	0.068					
CD14+ CD16+ monocyte %monocyte	MR-Egger	74.571	0.172	0.005	0.005	0.234	78.559	0.176
	IVW	76.255	0.160					
CD39+ CD8+ T cell %CD8+ T cell	MR-Egger	210.946	0.192	−0.003	0.002	0.115	215.553	0.156
	IVW	213.675	0.171					
CD3 on CM CD8+ T cell	MR-Egger	−0.003	0.348	−0.003	0.003	0.348	43.709	0.947
	IVW	41.985	0.954					

Assessing the heterogeneity of SNPs selected through Cochran's test, the results indicate that for Dendritic cell Absolute Count, CD62l- CD86+ myeloid Dendritic cell Absolute Count, CD62l- CD86+ myeloid Dendritic cell% Dendritic cell, CD14+ CD16+ monocyte% monocyte, CD39+ CD8+ T cell% CD8+ T cell, and CD3 on Central Memory CD4+ T cell, both IVW and MR-Egger's Q_pval are >0.05, suggesting the absence of heterogeneity. Additionally, the MR-PRESSO method did not identify any outliers (*P* > 0.05) (Table 2).

**Figure 4:**
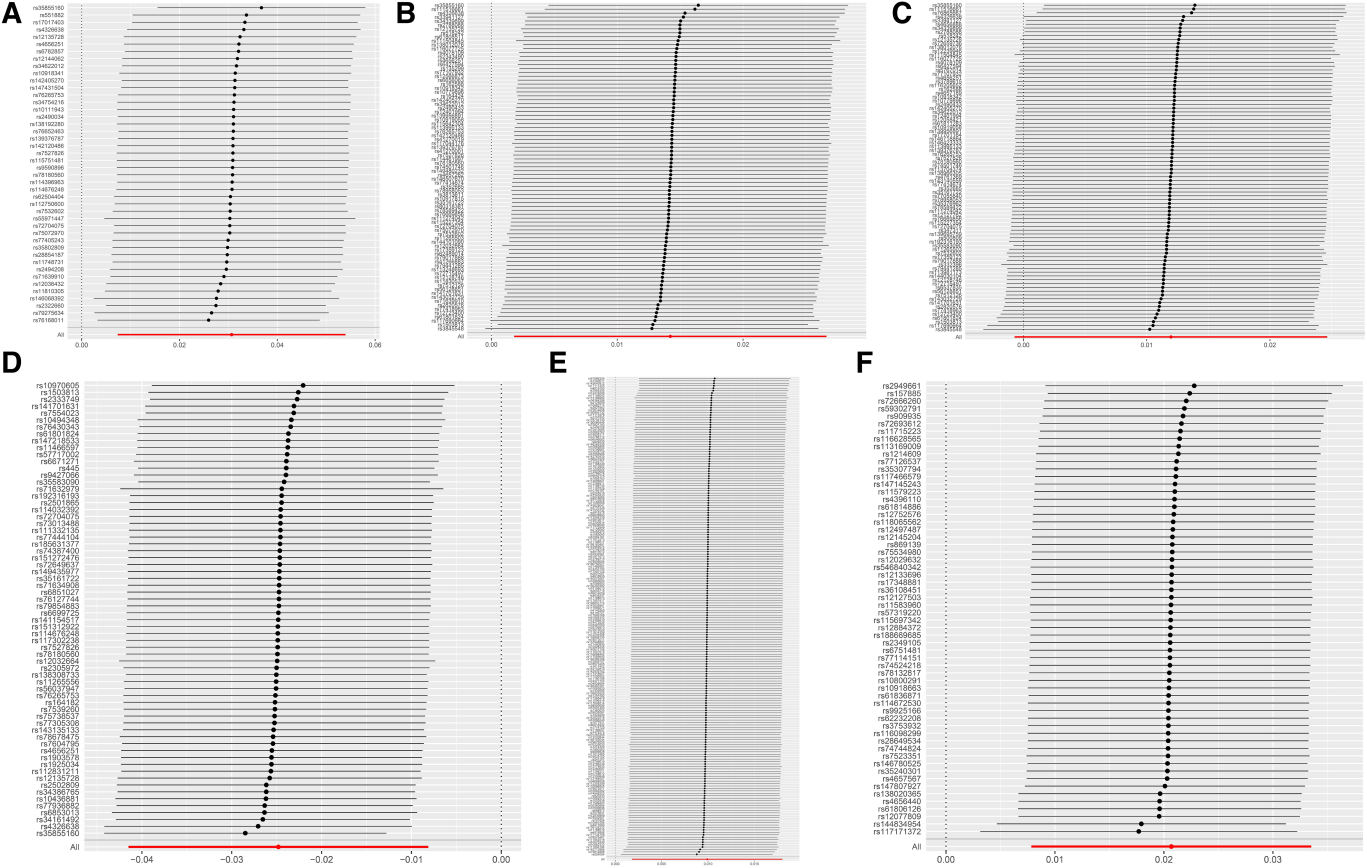
MR leave-one-out sensitivity analysis results. (**A**) DC Absolute Count. (**B**) CD62l- CD86+ MDC Absolute Count. (**C**) CD62l- CD86+ MDC %DC. (**D**) CD14+ CD16+ monocyte %monocyte. (**E**) CD39+ CD8+ T cell %CD8+ T cell. (**F**) CD3 on CM CD4+ T cell.

The leave-one-out analysis demonstrated a close alignment between the included effect size and the overall effect size, indicating the absence of a single SNP exerting a dominant influence on the overall assessment. This further enhances the stability and reliability of the MR statistics (Figure 4).

## 4 Discussion

Drawing upon an extensive dataset of publicly available genetic information, this study pioneered using a two-sample MR method to systematically assess the causal relationship between all discernible immune cell phenotypes and the overall incidence of HF. In the two-sample MR study, 38 immune cell-related factors were collectively identified as relevant to overall HF, including phenotypes associated with T cells, B cells, monocytes, and dendritic cells. Following stringent SNP quality control to mitigate confounding factors and reverse causation, we identified six immune cell-related exposure factors-Dendritic cell Absolute Count, CD62l- CD86+ Myeloid Dendritic cell Absolute Count, CD62l- CD86+ Myeloid Dendritic cell %Dendritic cell, CD14+ CD16+ Monocyte% Monocyte, CD39+ CD8+ T cell% CD8+ T cell, and CD3 on Central Memory CD4+ T cell-significantly associated with the overall risk of HF across three MR analysis methods (IVW, MR-Egger, WM). And the results of reverse MR showed no reverse causality between the six immune phenotypes and HF, suggesting that the effect of HF on these immune phenotypes was not statistically significant (*P* > 0.05).

Randomized controlled trial (RCT) stands as the gold standard in biomedical research for inferring causality (34). Nevertheless, RCTs are commonly hindered by their high costs, prolonged durations, and challenges in implementation, often stemming from ethical considerations and participant constraints. Observational studies, although more accessible for preliminary causation determination in etiological research, frequently encounter recognition challenges, especially when dealing with potential confounding factors and reverse causation (35). MR provides an effective solution to the aforementioned challenges by leveraging genetic variation as an instrumental variable. This approach adeptly mitigates confounding factors introduced by the environment. Furthermore, in contrast to the immediate outcomes derived from RCTs, exposure factors obtained from a genetic standpoint tend to endure over a lifetime and can even be hereditary to the next generation (36). HF, a chronic disease caused by multiple factors, remains poorly understood in its etiology despite extensive research into the mechanisms underlying its pathogenesis (37). Recent studies have revealed profound changes in the immune system during HF, encompassing the recruitment, activation of immune cells, and secretion of immune molecules induced by various factors. This intricate process of immune remodeling persists throughout the entire development and maintenance of HF (38). It is noteworthy that recent research emphasizes the significant impact of immune cells on the incidence, progression, and risk of HF (39, 40). Cells in the bloodstream, including neutrophils, macrophages, natural killer cells, eosinophils, and mast cells, demonstrate this effect by coordinating changes in tissue immune-inflammatory responses. This observation is corroborated by the upregulation of molecules such as PD-1 in regulatory T cells and oncostatin M in inflammatory macrophages. However, these observational studies only confirm the involvement of immune cells and inflammation in the pathogenesis of HF without providing reliable and significant causal evidence. Leveraging recent extensive GWAS on immune cell phenotypes and HF, two-sample MR studies provide a systematic evaluation of the causal relationship, offering an immunological perspective for clinical diagnosis and intervention in HF.

Dendritic cells, composed of diverse subsets from lymphoid and non-lymphoid organs, are specialized antigen-presenting cells that recognize pathogens in the innate immune system and activate immune cells in the adaptive immune system (41). Myeloid DCs and lymphoid DCs constitute the two primary sources of DCs, with myeloid DCs further categorized into precursor stage, immature DCs (iDCs), migratory phase, and mature DCs (mDCs) (42). The generation of autoimmune T cell responses to cardiac self-antigens requires direct interaction between antigen-presenting cells and T cells in lymphoid tissues; dendritic cells (DCs), as primary antigen-presenting cells, play a crucial role in regulating immune responses to foreign antigens and peripheral self-tolerance (43, 44). Our research uncovered a positive association between the immune phenotypes of Dendritic cell Absolute Count, CD62l- CD86+ Myeloid Dendritic cell Absolute Count, and CD62l- CD86+ Myeloid Dendritic cell% Dendritic cell, and the onset of HF. The upregulation of co-stimulatory molecule expression on the surface of DCs can activate T cells, inducing the occurrence of immune response and exacerbating the progression of HF (45). Furthermore, in studies related to myocardial ischemia, it has been observed that circulating DCs decrease in acute myocardial infarction patients due to their migration into myocardial tissue, confirming the involvement of DCs in the inflammatory processes of HF (46). MDCs (myeloid dendritic cells) are immune cells differentiated from myeloid stem cells under the stimulation of GM-CSF. Under different conditions, mDCs and iDCs (immature dendritic cells) can mediate the differentiation of CD+4 T cells into Th17 or Treg. This imbalance in the Th17/Treg cell ratio can lead to a close relationship with the imbalance of Th17 and Treg cells in the body, which is closely related to cardiac function (47–49). This suggests that mDCs play a crucial role in the immune mechanism of HF, and their involvement may accelerate the occurrence and progression of HF. Additional research has revealed that mDCs, by secreting a large amount of pro-inflammatory cytokine interleukin-12 (IL-12), promote the activation of T cells, leading to myocardial tissue remodeling and deterioration of heart function (50).

Monocytes, as a heterogeneous group of cells, are produced in the bone marrow, stored in the spleen, and circulate for 1–3 days in a balanced state (51). According to the expression of CD14, CD16 (Fc*γ* RIII), CD64 (Fc*γ* RI), and chemokine receptors CD192 and CX3CR1, peripheral blood monocytes can be classified into three distinct subgroups: Classical monocytes (CD14++CD16-): expressing high levels of CD14 and not expressing CD16; Intermediate monocytes (CD14+CD16+): expressing intermediate levels of both CD14 and CD16; Non-classical monocytes (CD14dimCD16+): expressing low levels of CD14 while expressing high levels of CD16 (52, 53). It is now acknowledged that classical monocytes (CD14++CD16-) mature along a continuum to intermediate monocytes (CD14++CD16+) and then to non-classical monocytes (CD14+CD16++) (54). Compared to the classical CD16- monocytes, the CD16+ subset possesses a stronger ability to secrete pro-inflammatory factors. Therefore, in many inflammatory diseases such as HF and tumors, there is an increase in the number of CD16+ monocytes, playing a role in eliminating damaging factors and protecting tissue cells to some extent (55). Rogacev and colleagues conducted a prospective follow-up study over an average of 4.9 years on 119 patients with chronic kidney disease. They found that the CD14++/CD16+ (intermediate) monocyte subset could predict combined mortality and major cardiovascular events (myocardial infarction, stroke, HF, vascular revascularization), independent of other risk factors, highlighting a significant correlation between the CD14+/CD16+ (intermediate) monocyte subset and the onset of HF (56). However, the article did not mention whether the authors discussed whether this correlation is positive or negative. The intermediate subset may represent a stage of differentiation towards immature classical and non-classical monocyte subsets (57). Benjamin J suggests that in both acute and chronic HF, the quantity and percentage of CD14+ CD16+ monocytes in patients increase, which correlates with the severity of the disease (58). Due to their potent phagocytic ability, CD14+ CD16+ monocytes play a crucial role in clearing apoptotic cells and endogenous ligands within the myocardium (59). Observational studies indicate that an increase in total monocyte count is associated with a poorer prognosis in HF (60). However, a lower count of CD14+ CD16+ monocytes is an independent predictor of increased mortality and repeated hospitalizations (58). This aligns with our conclusion: the percentage of CD14+ CD16+ monocytes among total monocytes is negatively correlated with HF. In terms of specific mechanisms, research has identified that CD14+ CD16+ monocytes possess a distinct gene expression profile, promoting angiogenesis and tissue remodeling through the upregulation of genes such as VCAM-1R (58, 61, 62). It is noteworthy that, although CD14++CD16+ monocytes exhibit dual functionality, being both pro-inflammatory and anti-inflammatory (63, 64), the CD14+ CD16+ intermediate subset effectively mitigates the harmful pro-inflammatory effects of classical and non-classical monocytes. This is achieved through pathways such as the production of interleukin-10 (IL-10), stimulation of angiogenesis, and promotion of tissue cell repair (65, 66).

The adaptive immune system's potential role in HF has been extensively explored, and T cells, crucial immune cells in adaptive immunity and chronic inflammation, participate in the development of HF. After maturing in the thymus, these cells enter the bloodstream and differentiate into two major subsets based on the surface expression of antigens: CD4+ and CD8+ T cells (67). Immunological basic theory research has revealed that the CD4+ subset includes effector T cells, involved in immune responses, and helper T cells, influencing antibody generation in cells. On the other hand, the CD8+ subset comprises suppressor cells, affecting B cell production, and cytotoxic cells, participating in the destruction of target cells (68). The interdependent interactions among cellular subsets in the body either promote or constrain each other, forming an appropriate immune response and maintaining the stability of the body's own immune system. According to the magnetic resonance (MR) results, we found that the proportion of CD39+ CD8+ T cells within CD8+ T cells is a significant factor in the onset of HF (*P* < 0.05). The CD39 molecule, a significant extracellular nucleotide hydrolase, is broadly expressed on endothelial and diverse immune cell surfaces (lymphocytes, neutrophils, monocytes/macrophages, dendritic cells, regulatory T cells, myeloid-derived suppressor cells, etc.), playing a critical role in immune responses, cell apoptosis, tumor immunity, and lymphocyte activation, emphasizing its crucial significance (69). Joe Yeong analyzed CD8+ T cells from healthy donors and found that only a minority of CD8+ T cells expressed CD39; however, during chronic infection, virus-specific CD8+ T cells exhibited significant CD39 expression. This suggests that CD8+ T cell expression of CD39 is a pathological phenomenon associated with the development of T cell exhaustion (70). Moreover, Mendel's randomized findings reveal a positive association between CD3 expression on Central Memory CD4+ T cells and the onset of HF. Specifically, each additional standard deviation of CD3 on Central Memory CD4+ T cells corresponds to a 2% increase in the risk of HF. Consequently, targeting the modulation of specific T cell subsets could represent a promising approach to enhance cardiac function, potentially slowing.

The early changes in immune cells are closely related to the pathology of HF. This suggests that we can develop more reliable immune-related biomarkers to aid in the early identification and diagnosis of HF. Additionally, various cells in the immune system participate in the body's inflammatory response, with peripheral immune cells such as monocytes, neutrophils, and T cells influencing the progression of central inflammatory responses through the release of pro-inflammatory factors. By modulating or intervening in specific immune cells, such as monocytes, dendritic cells, and T cells, we can slow down the emergence of early pathological features of HF, thus impeding its further development or reversing ventricular remodeling.

## 5 Strengths and limitations

Compared to previous studies, our study has several strengths. First, using data from immuno- phenotypic exposure factors and population HF studies has helped to mitigate biases arising from reverse causation and confounding factors. Second, we systematically sifted through a broad spectrum of 731 common immune phenotypes, delving deeper into the analysis of 6 exposure factors that exhibited significant causal relationships. Third, the causal relationships identified in our MR analysis could serve as promising candidates for further functional studies of immune phenotypes, contributing to the development of innovative approaches to target specific immune modulations and intervene in overall HF.

There are some limitations to the study we conducted. First, the study population was exclusively composed of individuals of European descent, emphasizing the need to further validate of the causal relationship between immune cells and relevant factors in the overall HF across diverse ethnic groups. Next, further stratified analysis of the population is not possible due to the lack of individual information. Third, the outcome data used in the study were aggregated, and the causal association between immune phenotypes and specific subtypes of HF is still unclear due to the lack of investigation of exposure to categorical diseases. Finally, this study solely assessed the causal association between immune cells and overall HF without delving into specific underlying mechanisms.

## 6 Conclusion

This study systematically assessed the causal effect of 38 immune cells and related factors on HF using a two-sample MR. The results identified potential causal relationships of specific immune phenotypes, such as Dendritic cell Absolute Count and CD62l- CD86+ Myeloid Dendritic cell, providing novel avenues for therapeutic exploration and insights into the pathogenic mechanisms of HF. However, these findings warrant further experimental validation and subsequent in-depth exploration of molecular mechanisms, laying the foundation for preventive and therapeutic strategies for all-cause HF and the potential discovery of drug targets.

## Data Availability

The original contributions presented in the study are included in the article/**supplementary material**, further inquiries can be directed to the corresponding authors.
